# Human inhibitory leukocyte Ig-like receptors: from immunotolerance to immunotherapy

**DOI:** 10.1172/jci.insight.151553

**Published:** 2022-01-25

**Authors:** Calvin D. De Louche, Ali Roghanian

**Affiliations:** Antibody and Vaccine Group, Centre for Cancer Immunology, School of Cancer Sciences, Faculty of Medicine, University of Southampton, Southampton General Hospital, Southampton, United Kingdom.

## Abstract

In recent decades, immunotherapeutic strategies have been used to treat a wide range of pathologies, many of which were previously incurable, such as cancer and autoimmune disorders. Despite this unprecedented success, a considerable number of patients fail to respond to currently approved immunotherapies or develop resistance over time. Therefore, there is an urgent need to develop the next generation of immune-targeted therapies. Various members of the Ig superfamily play essential roles in regulating leukocyte functions. One such group, the leukocyte Ig-like receptors (LILRs), have been implicated in both innate and adaptive immune regulation. Human inhibitory LILRs (LILRBs) are primarily expressed on leukocytes and mediate their signaling through multiple cytoplasmic immunoreceptor tyrosine-based inhibitory motifs. Engagement of LILRBs by endogenous and pathogenic ligands can markedly suppress immune responses, leading to tolerance or immunoevasion, whereas blocking these inhibitory receptors can potentiate immune responses. In this Review, we discuss the immunoregulatory functions of human LILRBs and the potential of targeting them to manipulate immune responses in various pathologies.

## Introduction

Immunotherapy regimens target and modulate the immune system to treat a pathology (1, 2). By harnessing the power of the body’s own immune system, various approved immunotherapeutic approaches either upregulate or downregulate the immune response to achieve beneficial outcomes (2). Along with widespread, publicized success with use in cancer therapies, the value of targeted immunotherapies may extend to infection (3), autoimmunity (4), and transplantation (5). Strategies that abrogate the immune response in autoimmune disorders and transplant recipients to inhibit overt immune activity, as opposed to attempts to amplify the immune response seen in malignancy and infection, may augment therapeutic outcomes (2, 3). Various treatments, including monoclonal antibodies (mAbs), chimeric antigen receptor (CAR) T cell therapies, vaccines, and immune checkpoint blockades, have been developed as immunotherapy modalities (6). Despite rapid uptake and unprecedented success of immunotherapy, many patients still do not respond to treatment and experience complex mechanisms of immune resistance or hypersensitivity (7). Thus, the development of the next generation of effective immune-targeted therapies that can provide stratified treatments and improve patient quality of life is required (7).

Ig superfamily members act as key regulators of leukocyte function (8). Leukocyte Ig-like receptors (LILRs; also called LIRs, ILTs, and CD85) are a family of 11 immunoregulatory receptors, encoded on chromosome 19 within the extended leukocyte receptor complex, that comprise two classes: activating LILRs (LILRA1–6) and inhibitory LILRs (LILRB1–5) (9, 10). LILRs were first identified in 1997 (11, 12) and were shown to share homology to the human killer cell inhibitory receptor (KIR) family, with notable parallels between the compositions of both their cytoplasmic domains and Ig-like structures (9). KIR expression is almost exclusively restricted to natural killer (NK) cells (13), while LILRs are found on most leukocyte subsets, including NK cells, T lymphocytes, B lymphocytes, and cells of the myeloid lineage — monocytes, macrophages, dendritic cells (DCs), and granulocytes (13). LILRs predominantly modulate the function of professional antigen-presenting cells such as macrophages, DCs, and B cells (11, 14, 15). Thus, these receptors are implicated in orchestrating innate and adaptive immune responses (11, 14–16). LILRBs are also expressed on a variety of other cells, such as osteoclasts (17) and endothelial and stromal cells (18), as well as cancerous cells, including leukemia subsets (9, 19, 20) (Table 1).

In particular, the LILRB subfamily present attractive therapeutic targets on which to focus next-generation immunotherapeutics (11, 12). LILRBs elicit inhibitory signaling via their long cytoplasmic tails that contain up to four immunoreceptor tyrosine-based inhibitory motifs (ITIMs) (9), which use the enzymatic phosphatase action of SHP1/2 proteins to suppress downstream signaling pathways, such as AKT and ERK, that normally promote effector function (9, 11, 15). LILRBs are typically overexpressed in cells traditionally associated with immunosuppression, such as immunosuppressive M2-skewed macrophages and tolerogenic DCs (21, 22). Multiple groups, including ours, have demonstrated that LILRB1 (also known as LIR1, ILT2, CD85j) ligation renders DCs tolerogenic, hindering the onset of adaptive immunity and promoting immunoevasion (21–24). In addition, both LILRB1 and LILRB2 (also known as LIR2, ILT4, CD85d) compete with CD8 for binding to the HLA class I molecule, inhibiting antigen-presenting cell activation and thereby altering downstream T cell responses (9). Further, interactions between both LILRB1 and LILRB2 and HLA-G present a potential mechanism for tumors and fetuses to overcome immunosurveillance and avoid immune attack (25, 26). Similarly, LILRB3 (also known as LIR3, ILT5, CD85a) ligation on human myeloid cells leads to upregulation of immunosuppressive genes associated with immunosuppressive M2 macrophages (27). Likewise, ligation of LILRB4 (also known as LIR5, ILT3, CD85k) and LILRB5 (also known as LIR8, CD85c) in macrophages regulates JAK/STAT signaling, mediating upregulation of immunosuppressive cytokines, such as IL-10, while downregulating inflammatory cytokines, such as IL-8 (28).

As such, ligation of LILRBs promotes tolerance and immunosuppression, whereas their antagonism induces an immunostimulatory milieu capable of generating potent immune responses (9). Here, we discuss and evaluate the potential of targeting LILRBs in different disease settings, where the alteration of LILRB function to modulate the immune response may prove beneficial.

## Cancer

The development of cancer is supported by immunoevasion mechanisms that include the production of antiinflammatory cytokines, induction of Tregs, and expression of immune checkpoint molecules (29). The term “immune checkpoint” refers to the network of inhibitory pathways that act to negatively regulate the magnitude of an immune response (30). These pathways minimize widespread tissue damage and maintain tolerance to self; however, tumors can modulate these inhibitory networks to promote immune cell exhaustion and resistance, which in turn fosters tumor proliferation and metastasis (30). This exploitation is achieved, in part, by the co-opting of immune checkpoints to suppress the recognition of tumor-associated antigens (TAAs) by T cell receptors (TCRs), thereby allowing tumors to avoid elimination (30). Programmed cell death protein 1 (PD-1) and cytotoxic T lymphocyte–associated antigen 4 (CTLA-4) are two well-studied inhibitory immune checkpoints that act as de facto off switches that limit T cell activation via ITIM signaling (30). Inhibition of aberrant checkpoint activity has been established as one of the most effective strategies to generate potent antitumor responses to date (31). Recent compelling evidence, as reviewed by Deng and colleagues (16), suggests that LILRBs function as key immune checkpoints during tumorigenesis that, unlike PD-1 and CTLA-4, show predominant expression on myeloid cell populations, rather than T cells (16). Therefore, targeting LILRBs may allow for the exploitation of myeloid-specific therapeutic pathways with the capacity to reduce tumor growth (16, 32, 33).

**Figure 1:**
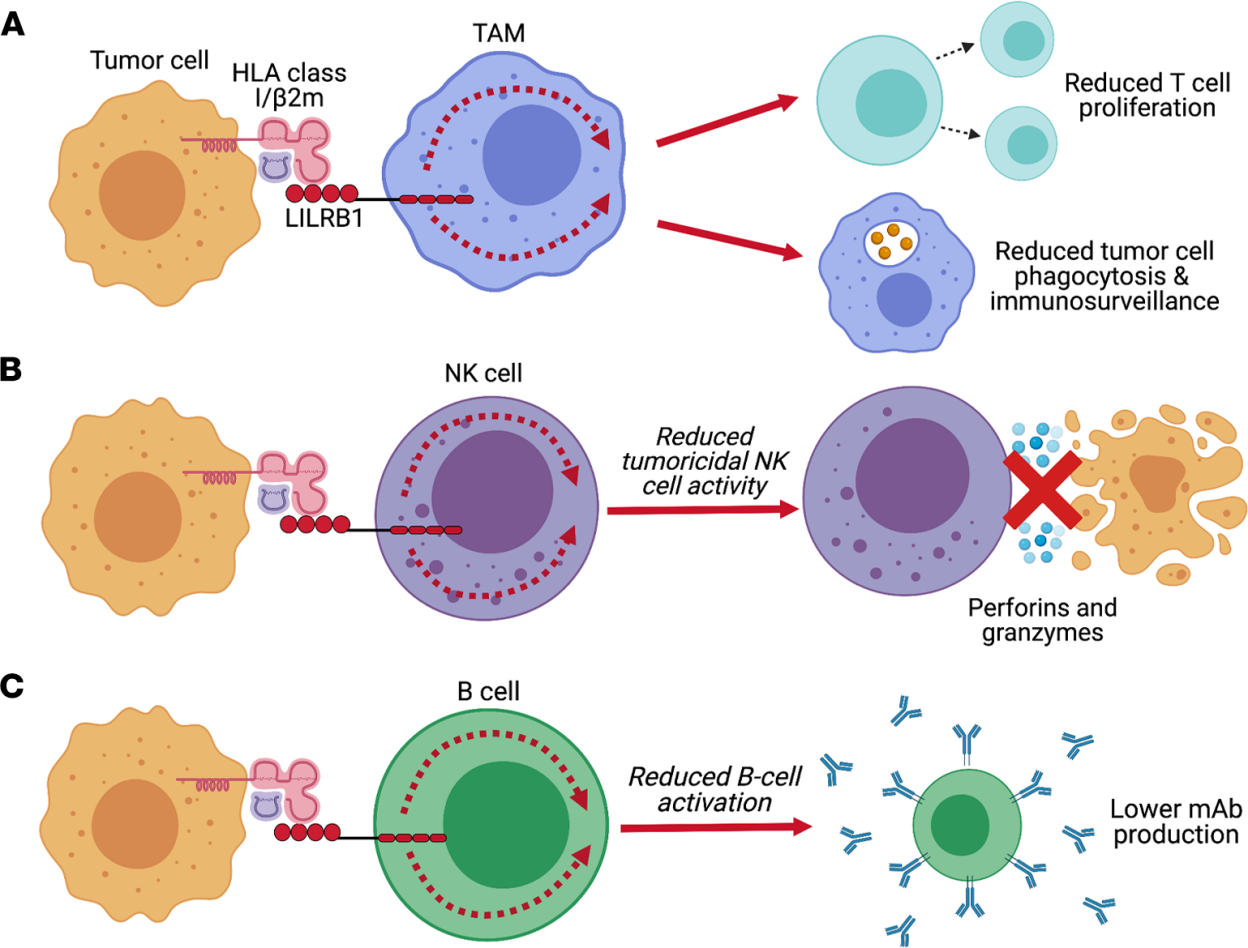
LILRB1-mediated regulation of myeloid and lymphoid cells. (**A**) Ligation of LILRB1 on tumor-associated macrophages (TAMs) by HLA class I/β_2_-microglobulin (β2m) expressed on tumor cells inhibits the phagocytic activity of TAMs, resulting in reduced immunosurveillance and enhanced tumor cell immunoevasion. (**B**) Peripheral NK cells from some patients with cancer express markedly high levels of LILRB1 molecules, which, upon engagement with HLA class I on tumor cells, leads to suppression of NK cell activity. Blocking this interaction with antagonistic mAbs has been experimentally shown to enhance the tumoricidal activity of NK cells against solid tumors and hematological malignancies. On the other hand, LILRB1-mediated inhibition of NK cells is an important mechanism by which overt immune responses in pregnancy may be controlled to avoid insult to the fetus. (**C**) Ligation of LILRB1 by HLA class I is capable of inhibiting B cell function, most notably reducing the secretion of auto/alloantibodies.

LILRB1 is expressed by CD8^+^ T cells and can inhibit T cell activation and proliferation (34–36). Moreover, LILRB1 controls thymocyte development and targets the proximal TCR signaling pathway (35). LILRB1 reduces the extent of TCR complex phosphorylation by inhibiting macromolecular assembly of two protein clusters (ZAP-70 and SLP-76) that are integral to the process (36). HLA class I is an endogenous LILRB1 and LILRB2 ligand and regulates macrophage effector function in the tumor microenvironment (TME; ref. 37) (Figure 1A). A recent study investigating HLA class I–LILRB1 interactions detailed a complex interplay between LILRB1 and β_2_-microglobulin (β2m) that implicates LILRB1 as a “don’t eat me” molecule (37, 38). As such, LILRB1 blockade (clone VMP55) augments tumor cell phagocytosis by macrophages, supporting the potential of modulating the HLA class I/LILRB1 signaling axis to promote antitumor immunity (37, 38). LILRB1 may also be considered as a diagnostic and prognostic target in gastric cancers (39), in addition to certain subtypes of adenocarcinoma (40). In adenocarcinoma, there is a positive association between LILRB1 expression and advanced pathological stage of malignancy (40). Moreover, peripheral NK cells from multiple myeloma (MM) and prostate cancer patients express markedly higher levels of LILRB1 when compared with those from healthy donors, which may hinder NK tumoricidal effects (41) (Figure 1B). Additionally, LILRB1 blockade (clone HPF1) on PBMCs from triple-negative breast cancer patients restored the cytotoxic function of NK cells (42). Similarly, LILRB1 blockade also increased the cytotoxic activity of NK cells against other solid and hematological malignancies (41, 43). HLA-G is another molecule known to be involved in the induction of immune tolerance, with its interaction with LILRB1 affecting B cell differentiation, proliferation, and antibody-secreting capacity (44, 45) (Figure 1C).

Eliciting tumor cell death by consolidating the cytotoxic action of CD8^+^ T cells represents an important mechanism by which to target cancer (46). Bispecific T cell engagers (BiTEs) concurrently engage both the TCR and TAAs (46). A recent study demonstrated that LILRB1 expression on CD8^+^ T cells inhibits priming by BiTEs, emphasizing LILRB1’s role in negatively regulating cytotoxic T cell function (46). Treatment with a LILRB1-blocking mAb (clone GHI/75) restored BiTE-mediated T cell activation (46). Intriguingly, previous studies have shown that LILRB1 loss in abnormal plasma cells may play a role in MM pathogenesis via a novel mechanism that allows early-stage malignancy to evade immune regulation (47–49). Lentiviral transduction was employed to force LILRB1 expression in human myeloma cell lines. Subsequent gene expression profiling of LILRB1-overexpressing cells showed notable downregulation of key MM pathogenesis–related genes. Moreover, LILRB1 overexpression in MM cells increased susceptibility to both T and NK cell–mediated killing (47). Here, the loss of LILRB1 may confer a survival advantage for malignant plasma cells (47). To date, research has focused on uncovering ways to antagonize LILRBs and reverse their inhibitory function to potentiate antitumor responses. These findings challenge the status quo and implicate LILRB1 agonism as an attractive therapeutic target with potential to restore the control exerted by LILRB1 in repressing the immune escape exhibited by early-stage MM (47).

Lung cancer is a leading cause of worldwide cancer mortality (50). Thus, the development of new therapeutics against novel immunotargets may improve patient prognosis (51). LILRB2 may be a suitable target, as increased LILRB2 expression in patients with lung adenocarcinoma correlates with reduced T cell infiltration in the tumor milieu, and is predictive of worsened clinical outcomes (51). Additionally, alongside promoting leukemia and blood stem cell development (52, 53), angiopoietin-related protein 2 can interact with LILRB2 to foster and promote the development of non–small cell lung cancer (NSCLC) (54–56). LILRB2 blockade markedly decreased not only NSCLC proliferation in culture, but also colony formation and migration (54). Furthermore, LILRB2 blockade in preclinical NSCLC models reduced granulocytic myeloid-derived suppressor cell (MDSC) and Treg infiltration and skewed tumor-associated myeloid cells toward a more inflammatory immune phenotype, with enhanced antitumor activity (57).

Interaction between LILRB2 and HLA-G promotes invasion, proliferation, and migration of colorectal cancer (CRC) via AKT and ERK signaling (58). Thus, antagonistic LILRB2 or HLA-G mAbs may prove efficacious against CRC and other tumors (58–61). More recently, tumor-derived LILRB2 was implicated in inducing T cell senescence (62). Thus, LILRB2 mediates a novel mechanism of immunosuppression in the TME, and may be a critical target for immune checkpoint–centered immunotherapies (62). Collectively, these observations strongly support the inhibition of LILRB2 signaling pathways as an important therapeutic target for mounting antitumor responses. Notably, an antagonistic LILRB2 mAb (clone MK-4830) has recently shown promise in a phase I clinical trial by demonstrating dose-related evidence of target engagement, while being well tolerated in patients with advanced solid tumors (ClinicalTrials.gov NCT03564691) (63, 64). Additionally, LILRB2 targeting using a different humanized mAb, JTX-8064, is currently in a first-in-human, dose escalation clinical trial in combination with anti–PD-1 inhibition (ClinicalTrials.gov NCT04669899) (65). If successful, these trials would represent a significant development in targeting LILRBs to modulate antitumor immune responses and may pave the way for the development of future therapeutics.

**Figure 2:**
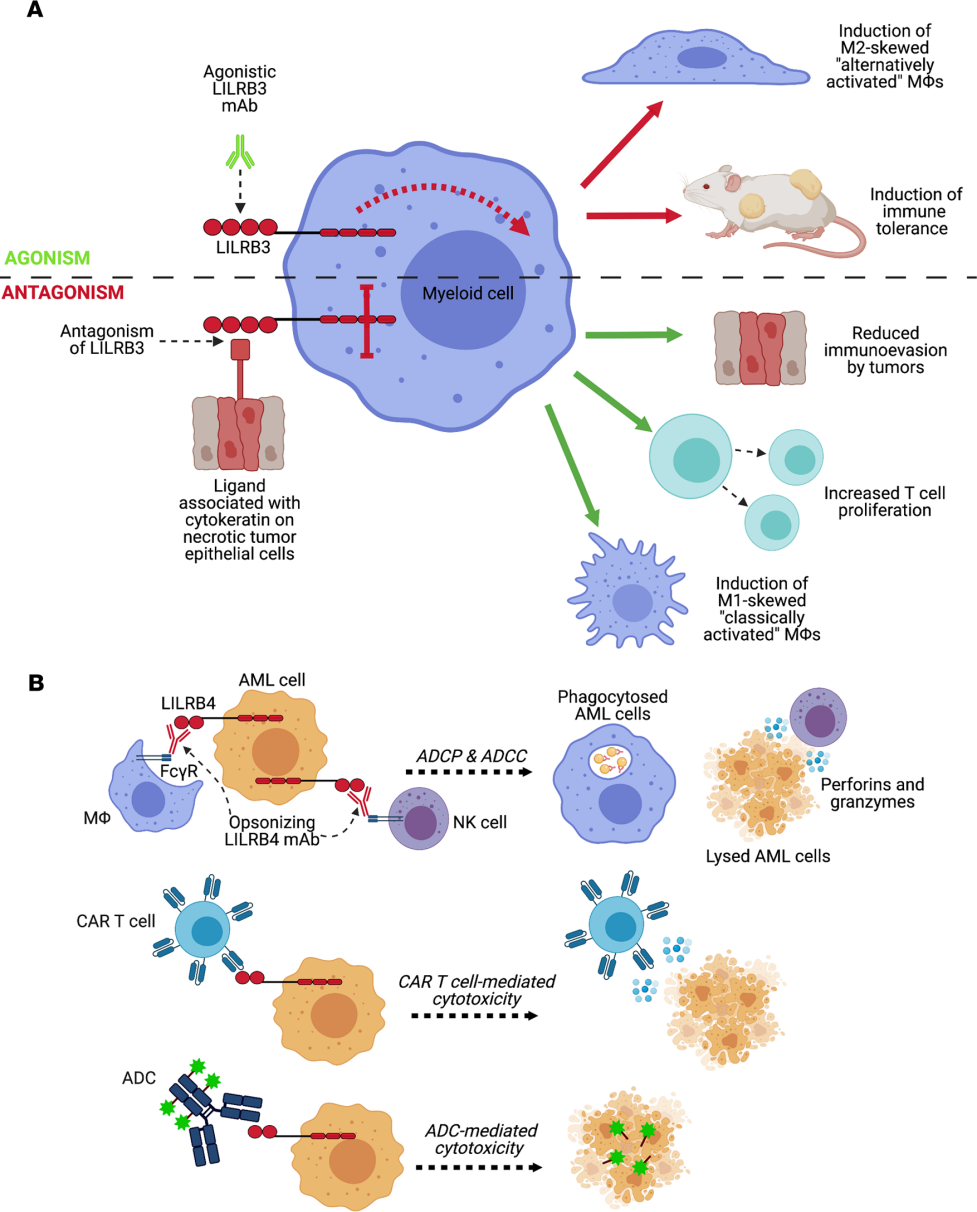
LILRB3- and LILRB4-mediated regulation of myeloid cells and their therapeutic potential. (**A**) Cross-linking of LILRB3 with agonistic mAb is capable of inducing both suppressive “M2-skewed” myeloid cells and immune tolerance. Therefore, ligating LILRB3 may have applications in both autoimmunity and transplant settings and could be especially useful in the transient induction of immune tolerance (top). Natural LILRB3 ligands expressed by necrotic cancer epithelial cells are able to induce inhibitory signaling through LILRB3, which is expected to lead to tumor immunoevasion. Thus, by modulating and disrupting the interaction between LILRB3 and its potential ligands, such extrinsic and intrinsic immunoevasion strategies may be prevented. This modulation would also allow both T cell proliferation and the induction of classically activated “M1-skewed” macrophages that are traditionally associated with an inflammatory milieu (bottom). (**B**) AML cell subsets overexpress LILRBs, including LILRB4. LILRB4 mAbs are capable of exerting potent anti-AML activity via activation of FcγRs on immune effector cells, such as macrophages and NK cells (top). Likewise, CAR T cells that target and bind LILRB4 epitopes with high affinity provide potent anti-AML capacity via T cell–mediated cytotoxicity (middle). Additionally, AML cells may be targeted for destruction by LILRB4-specific mAbs conjugated to toxins. Upon binding to LILRB4 and endocytosis by the target AML cells, the therapeutic toxic agent is internalized where it is subsequently capable of exerting a cytotoxic effect (bottom). ADC, antibody-drug conjugate; ADCC, antibody-dependent cellular cytotoxicity; ADCP, antibody-dependent cellular phagocytosis; AML, acute myeloid leukemia; CAR, chimeric antigen receptor; FcγR, Fcγ receptor; MΦ, macrophage.

Although the full immunomodulatory potential of LILRB3 has not yet been fully realized because of a lack of suitable reagents and preclinical models, it has been suggested that LILRB3 may interact with a cytokeratin-associated ligand expressed on necrotic glandular epithelial cells, including a number of human cancer cell lines (66) (Figure 2A). As induction of necrosis significantly impairs antigen-specific CD8^+^ T cell responses (67), ligation of myeloid LILRB3 by dying solid tumor cells is hypothesized to be a common immunoevasion mechanism within the TME (66). Disruption of the interaction between LILRB3 and its potential ligand on necrotic cancer cells may, thus, inhibit such intrinsic tumor immunoevasion strategies.

Tumor dissemination and metastasis are primary contributory factors to failure to respond to anticancer therapy and are responsible for 90% of all cancer-related deaths (68, 69). Like LILRB2, LILRB4 has been reported to control NSCLC pathogenesis, enhancing widespread NSCLC cell invasion and tumor angiogenesis, and may serve as an alternative strategy for NSCLC treatment (69). Blockade of LILRB4 expressed on monocytic MDSCs (M-MDSCs) reduces their ability to inhibit T cell responses (70). Therefore, LILRB4 antagonism may be useful in combatting immunosuppressive M-MDSC activity, while increasing T cell potency (70). Likewise, LILRB4 is moderately expressed by gastric cancers, and may contribute to inhibition of NK cell–mediated cytotoxicity (39). Soluble LILRB4 contributes to inhibition of T cell responses in solid tumors such as colorectal and pancreatic adenocarcinomas (71, 72). Treatment of solid tumors with an antagonistic LILRB4 mAb or serum depletion of LILRB4 restored previously repressed antitumor T cell responses, further implicating a supportive role for LILRB4 in tumor development (72). A recent study identified CD166 (also known as activated leukocyte cell adhesion molecule), expressed on activated T cells, as a ligand for LILRB4 (73). Subsequent knockdown of CD166 in human T cells eradicated the capacity for a LILRB4 fusion protein (LILRB4.Fc) to inhibit Th cell proliferation (73). Therefore, this ligand-receptor pair may act as an important immune checkpoint to target (72, 74).

Acute myeloid leukemia (AML) is characterized by the proliferation of abnormally differentiated myeloid cells, and ranks as the most common adult acute leukemia (75). Monocytic AML (M-AML) diagnosis often proves difficult owing to a lack of monocyte-specific markers. Coexpression of LILRB1 and LILRB4 has been identified as a highly specific marker capable of differentiating M-AML from non-monocytic AML (76). Thus, targeting LILRBs may provide an efficacious approach for developing M-AML treatments, specifically those using genetically engineered CAR T cells against certain LILRB epitopes (76, 77). In support of this, a LILRB4-targeting antibody (clone h128-3) was demonstrated to have potent anti-AML capability in preclinical models (78). Moreover, John and colleagues developed a CAR T cell that bound specifically to LILRB4 with high affinity and had potent effector function against AML cells in preclinical models (79) (Figure 2B). Importantly, no off-target toxicity was reported, and LILRB4 CAR T cells were capable of specifically targeting M-AML cells (79). More recent developments using LILRB4 antibody-drug conjugates (ADCs) show efficacious killing of LILRB4^+^ AML cells, without impacting normal progenitor cells (80). These data support LILRB4-targeting ADCs as a compelling strategy by which to eradicate M-AML cells, with potential to lead to safe drug candidates for future AML treatment (80) (Figure 2B). Mechanistically, LILRB4 has been shown to support leukemia cell migration and suppress T cell activity via activation of the ApoE/LILRB4/SHP2/uPAR/arginase-1 signaling axis in M-AML cells (81). A more comprehensive understanding of the specific ITIMs would prove useful for defining the mechanisms by which LILRBs regulate immune activity and tumor development (82). To this end, Zhang’s group identified the second (Y_412_) and third (Y_442_) ITIMs of LILRB4 as those responsible for facilitating signaling in M-AML cells, specifically for inhibiting downstream T cell proliferation (82). Further compelling evidence published by the same group demonstrated that LILRB3 expressed on AML cells recruits TRAF2 and cFLIP to stimulate NF-κB signaling, which enhances AML cell survival and impedes antitumor T cell activity (83). Subsequent LILRB3 blockade (clone no. 1NA) inhibited AML progression in vivo (83). Collectively, these studies demonstrate that targeting of LILRBs and their signaling pathways to treat AML has the potential to be more precise and efficacious than current approaches. In addition to enhancing AML cell deletion by phagocytic cells and NK cells, LILRB4 antagonism is expected to promote activation of T cells, subsequently enhancing their capacity to eliminate malignant cells (78, 83, 84). Notably, a LILRB4-blocking mAb (clone IO-202) is currently in a phase I clinical trial for the treatment of both AML and chronic myelomonocytic leukemia (ClinicalTrials.gov NCT04372433) (84).

## Infection

Pathogens evade the host immune system and establish chronic infection by modulating the immune response (85). Host and pathogen interactions are highly dynamic and involve signaling through complex intracellular pathways via either membrane-bound or cytosolic innate immune cell pattern recognition receptors, such as TLRs (86, 87). This, in turn, mediates production of effector molecules, including cytokines and antimicrobial peptides to combat the infective agent (88). The expanding LILRB field has begun to explore the importance of these receptors in immune dysregulation; however, little is known about their involvement in infection (89). As recently reviewed by Abdallah et al., a considerable number of pathogens are capable of interacting directly with LILRB family members to induce immunosuppression, which may prove especially pertinent during both ongoing and overt responses to infection (90). Conversely, blocking the interaction between LILRBs and pathogenic ligands may mitigate the immunoevasion that pathogens employ to prolong infection and perpetuate survival. While LILRBs have been shown to be involved during a number of viral, bacterial, and parasitic infections, their potential involvement in fungal infections remains unknown.

**Figure 3:**
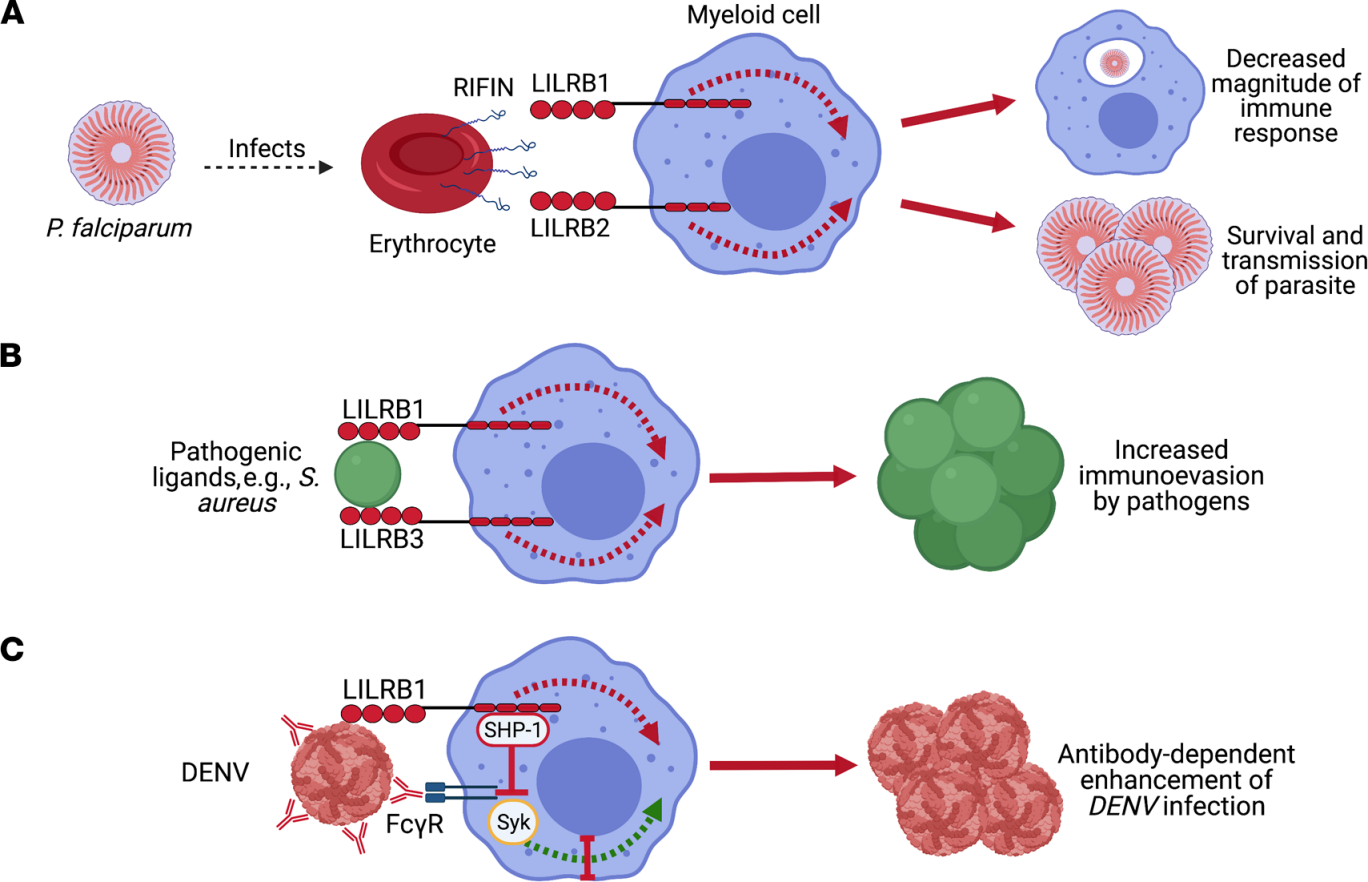
The interaction of pathogenic ligands with LILRBs promotes immunoevasion. (**A**) *P*. *falciparum infection* induces the expression of repetitive interspersed families of polypeptides (RIFINs) on the surface of infected erythrocytes. Binding of RIFINs to LILRB1 and LILRB2 is capable of decreasing the magnitude of the immune response and supports the continued survival of the parasite in the host. (**B**) Putative pathogenic ligands, such as those expressed by *S*. *aureus*, have been shown to engage LILRB1 and LILRB3, which may potentially help them evade the immune responses. (**C**) Dengue virus (DENV) is capable of binding LILRB1 and inducing its inhibitory signaling pathways to evade the early antiviral immune response. Engagement of LILRB1 by DENV inhibits IFN-stimulated genes and reduces FcγR-mediated phagocytosis of opsonized DENV particles.

The malaria parasite *Plasmodium falciparum* (*P.* *falciparum*) is one of the deadliest in humans (91). Once inside host erythrocytes, *P.* *falciparum* generates repetitive interspersed families of polypeptides (RIFINs) that are expressed at the surface of infected erythrocytes (91, 92). RIFINs were recently shown to bind to LILRB1 and LILRB2 by mimicking the structure of HLA class I (92, 93) (Figure 3A). Thus, *P.* *falciparum* uses molecular mimicry to initiate ITIM-mediated signaling in immune cells, potentially dampening the magnitude of the immune response and facilitating parasite survival and transmission (92–94). Interestingly, a single point mutation in RIFIN abolishes LILRB1 binding (92). Such point mutations could be useful in the context of resensitizing the immune system to respond to subsequent malaria infection.

Bacteria, such as *S. aureus*, are believed to interact with LILRB2 and LILRB3; however, the functional consequences of these interactions remain unknown (95) (Figure 3B). Furthermore, LILRB5 expression is elevated in *Mycobacterium-*exposed monocytes, but there have not been any investigations into whether this upregulation influences the immune response (96). Bacterial components of *Salmonella typhimurium* (*S.* *typhimurium*), namely LPS and flagellin, trigger TLRs. Infection of macrophages with *S.* *typhimurium* markedly upregulated LILRB2 and LILRB4 (89). Moreover, LILRBs and TLRs are expressed on similar cell types and have profound effects on each other’s expression and function. LILRB ligation inhibits TLR function, while TLR activation modulates LILRB expression (89). Interplay between LILRBs and TLRs may therefore represent a fine balancing act, with LILRB upregulation in response to TLR activation providing a mechanism by which overt immune responses may be controlled (89). LILRB binding to exogenous ligands may additionally help pathogens evade immune attack (89).

The host response to sepsis is complex and involves conflicting processes of overt inflammation and immune suppression (97, 98); however, the precise mechanisms underpinning these interactions remain unclear (97). To this end, LILRB3 is markedly upregulated in sepsis patients’ PBMCs (97). LILRB3 can inhibit antigen presentation by macrophages, impeding Th1 immune responses. Furthermore, treatment with a peptide that antagonized paired Ig-like receptor B (PIR-B), the murine LILRB ortholog, augmented survival of septic mice with pulmonary pathology, implicating LILRB3 as a potential target to treat sepsis (97). Similarly, McCarthy’s group recently demonstrated that LILRB3 ligation on neutrophils markedly inhibits key IgA-mediated effector function, including microbial killing and phagocytic uptake (99). In this context, LILRB3 acts as a critical checkpoint to control activation of human neutrophils. Thus, LILRB3 modulation may present another point of intervention for fine-tuning the immune response during active infection (99).

DCs ubiquitously express LILRBs and primarily help initiate, but also regulate, the immune response against pathogens (23). Continuous LILRB1 ligation on DCs by a LILRB1 mAb (clone HPF1) or UL18-Fc (a natural human cytomegalovirus-derived LILRB1 ligand) renders DCs tolerogenic, with poor T cell stimulatory capacity that continues after exposure to bacterial LPS (23). These findings implicate LILRB1 as an important mediator in maintaining the fine balance between the induction and suppression of adaptive immunity and may permit intervention to modulate the immune response to infection (23). On the other hand, coligation of LILRB4 with FcγRI on THP-1 cells inhibits FcγR-dependent uptake of antibody-opsonized bacterial particles via marked LILRB4-dependent dephosphorylation of key signaling proteins, including clathrin, SYK, and the E3 ubiquitin protein ligase CBL (100). These results highlight LILRB4 as a regulator of signaling molecules involved in FcγRI-mediated clathrin-dependent endocytosis and phagocytosis of bacterial pathogens (100).

Similarly to parasites and bacteria, viruses also seek to evade host immune responses in order to replicate (101). Dengue virus (DENV) evades the early antiviral immune response by binding LILRB1, which, via ITIM signaling, attenuates lysosomal enzyme activation (102), inhibits FcγRs, and reduces expression of IFN-stimulated genes (101) (Figure 3C). Therefore, inhibiting DENV-LILRB1 interactions presents a potential strategy for vaccines or other immunotherapeutics against the virus. Patients capable of maintaining undetectable levels of HIV-1 replication in the absence of antiretroviral therapy (elite controllers) have circulating DCs with increased antigen-presenting capability and diminished capacity to secrete proinflammatory cytokines (103). This unique immune profile is associated with, and maintained by, a notable upregulation of LILRB1 and LILRB3 (103). Importantly, this specific functional profile may protect patients from excessive HIV-1–related immune activation while initiating memory T cell responses (103). These findings uncover previously unrecognized aspects of immune protection against HIV-1 in elite controllers, and present a novel outlook for manipulating host immunity against HIV-1, either prophylactically or after disease onset (103). Additionally, S100A9-mediated ligation of LILRB1 can stimulate potent anti–HIV-1 activity of NK cells (104). This S100A9-LILRB1 interaction is hypothesized to be important in DC–NK cell crosstalk and may influence formation of specific antiviral immune responses (104). Moreover, the human cytomegalovirus (CMV) HLA class I homolog UL18 interacts with LILRB1 and is thought to assist CMV in evading the immune system (105). However, UL18-LILRB1 interaction remains controversial owing to paradoxical findings indicating that UL18 inhibits LILRB1^+^ cells but activates LILRB1^–^ NK cells (106, 107). Furthermore, interaction between UL18 and LILRB1 on CD8^+^ T cells may be important in controlling the scale of CMV episodes (107). Both resting and active CD8^+^ T cells are capable of lysing CMV-infected UL18-expressing cells, but not UL18-deficient cells, with lysis blocked by antagonistic LILRB1 and UL18 mAbs (107). The precise details of UL18 involvement in CMV infection remain unknown; however, LILRB1 may still present a useful biomarker for human CMV infection, as its upregulation correlates with CMV reactivation after lung transplantation (108).

## Autoimmunity

Autoimmunity occurs in 3%–5% of the population and is driven by both genetic and environmental influences (109). A triad of factors encompassing the overall reactive state of the immune system, the specific autoantigen, and the targeted tissue contribute to autoimmune susceptibility (109). Autoimmune disorders pose a significant clinical problem, due to their chronic nature and health care system burden (110). As such, it is vital that effective treatment strategies are implemented. Indeed, TNF-α antagonists have elicited great success in rheumatoid arthritis (RA), and various other immunotherapies have shown notable efficacy (111, 112). Despite this, most therapeutic regimes only target the resulting terminal inflammation and fail to address key issues that underpin initial autoimmunity development (110). Hence, we require an understanding of how abnormal immune responses arise, but also of any inherent mechanisms that exist to suppress these responses. LILRBs are implicated in autoimmunity and have the potential to be exploited to suppress such pathologies. Furthermore, LILRBs may help orchestrate autoimmunity pathogenesis, as certain polymorphisms and deletions in LILRB-encoding genes have been associated with autoimmune disorders and Treg generation (113). Importantly, agonizing LILRBs to induce tolerance and anergy may present a highly effective strategy to combat autoimmune disorders.

Multiple sclerosis (MS) is a chronic autoimmune disease of the CNS that leads to axonal damage, neuron demyelination, and chronic inflammation (114). Murine experimental autoimmune encephalomyelitis (EAE) is an MS model generated by immunization of mice with myelin oligodendrocyte glycoprotein (MOG) (114). In this model, LILRB4.Fc was shown to bind to CD166 on activated CD4^+^ and CD8^+^ T cells and subsequently limited disease evolution by inhibiting production of proinflammatory cytokines, such as IFN-γ and IL-17A, that contribute to neuroinflammation and paralysis (114). LILRB4.Fc-treated mice exhibited reduced inflammatory infiltrate and fewer demyelinated areas in both the brain and spinal cord (114). Moreover, LILRB4.Fc administration, via reverse signaling through its ligand (CD166), markedly reduced proliferation of MOG-specific Th1 and Th17 cells and ameliorated the effects of EAE (114). In the context of LILRB-targeting immunotherapies, inhibition of Th1 and Th17 cell development may provide clinical benefit for individuals with MS (114). Glatiramer acetate (GA) is approved for therapeutic use in patients with relapsing-remitting MS (115). The mechanism of action of GA is not entirely clear; however, it stimulates the release of antiinflammatory cytokines such as IL-10 and TGF-β, promotes Th2 immunity, and increases M-MDSC populations (115). GA was recently demonstrated to associate directly with both LILRB2 and LILRB3, suggesting a potential mechanism by which it exerts its immunosuppressive effect (115).

Inhibition of B cell function, specifically antigen presentation and antibody secretion, is a crucial strategy in targeting B cell–mediated autoimmune disorders (44). LILRB1 is the only LILRB member highly expressed on B cells and is known to interact with HLA-G to induce immunotolerance by inhibiting B cell function (44). Therefore, strategies to disrupt this interaction may provide an avenue for B cell–targeted therapies that aim to limit autoimmune responses (44, 45). Interestingly, circulating HLA-G has been suggested to be increased in plasma of patients with chronically inflamed RA but is not recognized by LILRB1 (116). In this instance, LILRB1 is unable to exert its immunosuppressive effects against chronic inflammation (116). HLA-G can form dimers, which greatly augments its recognition by both LILRB1 and LILRB2, but the conditions under which dimerization occurs are not well understood (117). Increased levels of HLA-G monomers or other non-classical–like structures (118) may explain why elevated HLA-G in certain patients fails to correlate with subsequent recognition by LILRB1 (116).

Systemic lupus erythematosus (SLE) primarily affects the skin, joints, brain, blood vessels, and serous membranes (119, 120). Patients with SLE display immunological differences, including altered T cells, polyclonally activated B lymphocytes, and high levels of various autoantibodies (121, 122). B cell hyperactivity is of particular interest and, despite being of unknown cause, is postulated to result from defective immune regulation (119). Monsiváis-Urenda and colleagues demonstrated that SLE patient PBMCs exhibited poorly functioning LILRB1, with B lymphocytes from the same patients displaying diminished receptor expression (119). This dysfunctional LILRB1 in patients with SLE may contribute to disease development via loss of immune regulation (119). Moreover, circulating plasmacytoid DCs are elevated in patients with SLE and have reduced LILRB1 expression, which correlates with disease severity (119, 123). Therefore, therapies (e.g., antiinflammatory cytokines) that enhance and/or agonize LILRB expression on leukocytes, with the aim of reducing both the array and severity of autoimmune symptoms in patients with SLE, are of great interest (123).

LILRB1 polymorphisms may also predispose individuals to autoimmune disorders and play an active role in disease pathogenesis (10). In this regard, certain LILRB1 polymorphisms, specifically the LILRB1-PE-01/01 genotype, have been shown to associate with reduced susceptibility and disease severity in patients with RA (124). Conversely, LILRB2 is markedly downregulated on monocytes from patients with psoriatic arthritis and may contribute to disease progression (125). Hence, modulating LILRB2 expression on human monocytes may reverse psoriatic arthritis–associated effects (125). In comparison with LILRB1 and LILRB2, LILRB3 is highly polymorphic (10, 11). As the extracellular domains of both LILRB3 and LILRA6 are highly homologous, it has been difficult to genetically and functionally differentiate the two, precluding disease association studies (126). More recently, notable variations between LILRB3 and LILRBA6 have been found at both the DNA and protein levels in a Japanese population (126), thus serving as a potential foundation for future disease association studies (126). A GWAS involving both Turkish and North American cohorts identified genetic susceptibility loci in *RPS9*/*LILRB3* (rs11666543) for the rare inflammatory disease Takayasu arteritis, which predominantly affects the aorta and manifests with arterial stenosis and progressive occlusion (127, 128). The risk allele was associated with significant reduction in LILRB3 expression, implying that reduced LILRB3-mediated inhibitory signaling results in an unbridled immune response that contributes to disease pathogenesis (127). These findings provide additional insight into the genetic associations that underpin immunopathogenesis of chronic autoimmune conditions but also suggest potential avenues to treat such conditions via targeting of LILRBs and/or their ligands (116).

## Transplantation

Transplantation procedures generate both thermal and metabolic stresses that increase the magnitude of immune response against non–self-antigens expressed on donor tissues (129). This leads to widespread inflammation that generates a potent immune response against the graft tissue, resulting in either acute or chronic graft rejection (129, 130). Over the past five decades, significant advances have been made in the development of immunotherapeutic agents that are capable of reducing graft rejection among transplant patients (130). Earlier studies in transgenic mice have shown that LILRB1 or LILRB2 expression on host myeloid cells can prolong graft survival (35, 60, 131). This improved graft survival was a result of substantial T cell suppression and in vivo expansion of MDSCs and Tregs (35, 60, 131). As such, LILRBs present an exciting target that may be exploited with novel immunotherapeutic agents after transplantation. For example, LILRB agonism with soluble HLA-G may elicit immunosuppressive effects and thereby suppress the immune system during transplantation. Here, parallels may be drawn to interactions between HLA-G and LILRB1/LILRB2 in pregnancy, that limit potentially harmful overt maternal immune responses against the developing fetus by impairing NK cell cytotoxicity and inhibiting both T and B cell proliferation (132). Through further exploration of mechanisms to agonize LILRBs and exploit their immunosuppressive, graft-sparing properties, LILRB-specific therapies that reduce the incidence of graft rejection and ameliorate the prognoses of patients undergoing transplantation may be developed.

Using a reconstituted humanized mouse model, we recently demonstrated that in vivo LILRB3 ligation with an agonistic mAb (clone A1) prior to engraftment of allogeneic lymphoma cells induced tolerance (27) and enabled donor cell engraftment (133) (Figure 2A). The improved tolerance was due to reprogramming of myeloid cells toward a more suppressive phenotype with capacity to suppress T cell responses. Therefore, LILRB3 ligation highlights a potential strategy to exploit the immunoregulatory effects of the receptor to induce transient immune tolerance (27). Likewise, ligation of LILRB4 has been shown to confer significant immunoregulatory functions in the transplantation setting (28). Importantly, induced expression of LILRBs on immune cells can increase tolerogenic capacity. For example, DCs transduced with a LILRB4-carrying lentiviral vector had reduced ability to stimulate proliferation of allogeneic T cells and had increased capacity to induce CD4^+^CD25^+^Foxp3^+^ Tregs in vitro (134). While this approach still requires in vivo validation, it may be pivotal for developing therapies that promote allograft survival in transplant patients (134). Another study demonstrated that LILRB4.Fc-treated diabetic humanized mice tolerated transplanted allogenic pancreatic islet cells and maintained good blood sugar control, whereas control mice rejected the graft (135). LILRB4.Fc treatment reduced proinflammatory cytokine expression and induced T suppressor cells (135). Further work using NOD/SCID mouse models of xenogeneic graft-versus-host disease (GVHD) revealed that LILRB4.Fc treatment inhibited both cellular and humoral aspects of GVHD pathogenesis (136). Specifically, LILRB4.Fc prevented the maturation of both helper and cytotoxic T cells and instead induced Tregs, which help to maintain long-term tolerance (137). Moreover, LILRB4-induced T suppressor cells showed significant upregulation of zinc finger proteins, transcriptional repressors that play a critical role in further differentiation of LILRB4-induced T suppressor cells (138).

HLA-G is associated with induction of tolerance and positively correlates with improved outcomes in transplantation expression during allogeneic recognition (131). As such, HLA-G–expressing antigen-presenting cells are capable of inhibiting T cell alloproliferation by binding to LILRB1 and LILRB2 with high affinity, thereby promoting graft acceptance (139). Furthermore, LILRB1 engagement with HLA-G has the capacity to expand MDSC populations and increase graft survival time in allogeneic skin transplant recipients (131). As outlined previously, HLA-G binding to LILRB1 also suppresses B cell responses, which may have use in minimizing humoral responses in transplantation (44).

The highly polymorphic nature of LILRB3 has also been implicated in transplantation and GVHD onset in patients with transplanted hematopoietic stem cells (HSCs). As such, alterations in the LILRB3 amino acid sequence between recipients and donors permit the production of alloantibodies (140). One study reported that 5.4% of HSC transplant recipients had LILRB3-targeting antibodies that may promote a graft-versus-leukemia effect against LILRB3-expressing leukemic cells, proposing LILRB3 as a viable target for antibody-based immunotherapies (140).

## Discussion

There has been rapid uptake in the use of immunotherapeutic strategies to treat an array of pathologies in recent decades. Many patients still fail to respond to approved therapies, and therefore development of new treatment regimens that benefit all disease categories, whether by amplifying or abrogating the immune response, is mandated. The LILRB field continues to expand, and the adaptable nature of targeting of LILRBs to combat a plethora of immune-related pathologies highlights these receptors as viable immunotherapeutic targets. The potential for LILRBs to potently modulate the immune response has enticed many academic groups and pharmaceutical companies to develop novel therapeutics that target this key receptor family. A few clinical trials investigating the safety and efficacy of LILRB-targeting mAbs in patients with cancer are well underway and, if successful, will change the face of LILRB targeting to potentiate efficacious and safe antitumor immune responses. Moreover, further evaluation and understanding of endogenous LILRB ligands may be useful for developing agents capable of ligating these receptors to reduce the effects of autoimmune disease and graft rejection. However, given the wide expression of LILRBs on various immune and some nonimmune cells, extra precautionary measures should be taken when developing LILRB-targeting agents. For instance, LILRB2 is reportedly expressed on platelets, increasing the likelihood of off-target toxicities associated with any therapeutics against this receptor (141). Furthermore, LILRB2 is expressed by neurons and has been suggested to be implicated in the dysregulation of the CNS (142).

In summary, by exploiting the opportunity for immune intervention provided by LILRBs, we look, with optimism, to a future where novel immunotherapeutic agents that manipulate LILRB-mediated immunoregulatory functions are commonplace. From the laboratory bench, to forming an integral part of frontline therapy against key immune-related pathologies at the bedside, such therapeutics may improve patient prognoses and quality of life.

## Figures and Tables

**Table 1:**
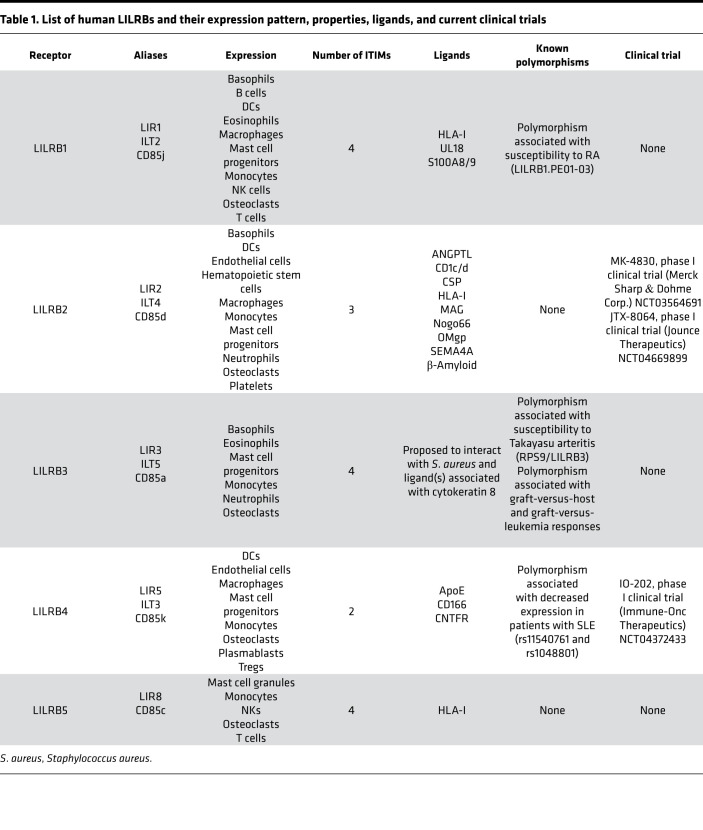
List of human LILRBs and their expression pattern, properties, ligands, and current clinical trials

## Version 1 01/25/2022

Electronic publication

## References

[1] Naran K (2018). Principles of immunotherapy: implications for treatment strategies in cancer and infectious diseases. Front Microbiol.

[2] Wraith DC (2017). The future of immunotherapy: a 20-year perspective. Front Immunol.

[3] Bucktrout SL (2018). Recent advances in immunotherapies: from infection and autoimmunity, to cancer, and back again. Genome Med.

[4] [No authors listed] (2019). Immunotherapies for autoimmune diseases. Nat Biomed Eng.

[5] Koura DT (2013). In vivo T cell costimulation blockade with abatacept for acute graft-versus-host disease prevention: a first-in-disease trial. Biol Blood Marrow Transplant.

[6] Mellman I (2011). Cancer immunotherapy comes of age. Nature.

[7] Bai R (2020). Mechanisms of cancer resistance to immunotherapy. Front Oncol.

[8] Farré D (2017). Immunoglobulin superfamily members encoded by viruses and their multiple roles in immune evasion. Eur J Immunol.

[9] Van Der Touw W (2017). LILRB receptor-mediated regulation of myeloid cell maturation and function. Cancer Immunol Immunother.

[10] Hirayasu K, Arase H (2015). Functional and genetic diversity of leukocyte immunoglobulin-like receptor and implication for disease associations. J Hum Genet.

[11] Colonna M (1997). A common inhibitory receptor for major histocompatibility complex class I molecules on human lymphoid and myelomonocytic cells. J Exp Med.

[12] Samaridis J, Colonna M (1997). Cloning of novel immunoglobulin superfamily receptors expressed on human myeloid and lymphoid cells: structural evidence for new stimulatory and inhibitory pathways. Eur J Immunol.

[13] Katz HR (2006). Inhibition of inflammatory responses by leukocyte Ig-like receptors. Adv Immunol.

[14] Colonna M (1998). Human myelomonocytic cells express an inhibitory receptor for classical and nonclassical MHC class I molecules. J Immunol.

[15] Nakajima H (1999). Human myeloid cells express an activating ILT receptor (ILT1) that associates with Fc receptor gamma-chain. J Immunol.

[16] Deng M (2021). Leukocyte immunoglobulin-like receptor subfamily B: therapeutic targets in cancer. Antib Ther.

[17] Mori Y (2008). Inhibitory immunoglobulin-like receptors LILRB and PIR-B negatively regulate osteoclast development. J Immunol.

[18] Kim-Schulze S (2006). Regulation of ILT3 gene expression by processing of precursor transcripts in human endothelial cells. Am J Transplant.

[19] Volz A (2001). Genesis of the ILT/LIR/MIR clusters within the human leukocyte receptor complex. Immunol Rev.

[20] Kang X (2016). Inhibitory leukocyte immunoglobulin-like receptors: Immune checkpoint proteins and tumor sustaining factors. Cell Cycle.

[21] Chang CC (2002). Tolerization of dendritic cells by T(S) cells: the crucial role of inhibitory receptors ILT3 and ILT4. Nat Immunol.

[22] Banchereau J (2012). Immunoglobulin-like transcript receptors on human dermal CD14+ dendritic cells act as a CD8-antagonist to control cytotoxic T cell priming. Proc Natl Acad Sci U S A.

[23] Young NT (2008). The inhibitory receptor LILRB1 modulates the differentiation and regulatory potential of human dendritic cells. Blood.

[24] Brenk M (2009). Tryptophan deprivation induces inhibitory receptors ILT3 and ILT4 on dendritic cells favoring the induction of human CD4+CD25+ Foxp3+ T regulatory cells. J Immunol.

[25] Sheu J, Shih Ie M (2010). HLA-G and immune evasion in cancer cells. J Formos Med Assoc.

[26] Kuroki K, Maenaka K (2007). Immune modulation of HLA-G dimer in maternal-fetal interface. Eur J Immunol.

[27] Yeboah M (2020). LILRB3 (ILT5) is a myeloid cell checkpoint that elicits profound immunomodulation. JCI Insight.

[28] Liu J (2020). LILRB4, from the immune system to the disease target. Am J Transl Res.

[29] Dyck L, Mills KHG (2017). Immune checkpoints and their inhibition in cancer and infectious diseases. Eur J Immunol.

[30] Pardoll DM (2012). The blockade of immune checkpoints in cancer immunotherapy. Nat Rev Cancer.

[31] Rotte A (2019). Combination of CTLA-4 and PD-1 blockers for treatment of cancer. J Exp Clin Cancer Res.

[32] Zhang J (2017). Leukocyte immunoglobulin-like receptors in human diseases: an overview of their distribution, function, and potential application for immunotherapies. J Leukoc Biol.

[33] Fan J (2021). Expression of leukocyte immunoglobulin-like receptor subfamily B expression on immune cells in hepatocellular carcinoma. Mol Immunol.

[34] Gustafson CE (2017). Immune checkpoint function of CD85j in CD8 T cell differentiation and aging. Front Immunol.

[35] Liang S (2006). Human ILT2 receptor associates with murine MHC class I molecules in vivo and impairs T cell function. Eur J Immunol.

[36] Purbhoo MA (2010). Dynamics of subsynaptic vesicles and surface microclusters at the immunological synapse. Sci Signal.

[37] Barkal AA (2018). Engagement of MHC class I by the inhibitory receptor LILRB1 suppresses macrophages and is a target of cancer immunotherapy. Nat Immunol.

[38] Zhao J (2019). The MHC class I-LILRB1 signalling axis as a promising target in cancer therapy. Scand J Immunol.

[39] Zhang Y (2012). Expression of immunoglobulin-like transcript (ILT)2 and ILT3 in human gastric cancer and its clinical significance. Mol Med Rep.

[40] Cheng J (2020). Leukocyte immunoglobulin-like receptor subfamily B member 1 potentially acts as a diagnostic and prognostic target in certain subtypes of adenocarcinoma. Med Hypotheses.

[41] Chen H (2020). Antagonistic anti-LILRB1 monoclonal antibody regulates antitumor functions of natural killer cells. J Immunother Cancer.

[42] Roberti MP (2015). Overexpression of CD85j in TNBC patients inhibits cetuximab-mediated NK-cell ADCC but can be restored with CD85j functional blockade. Eur J Immunol.

[43] Guillerey C (2016). Targeting natural killer cells in cancer immunotherapy. Nat Immunol.

[44] Naji A (2014). Binding of HLA-G to ITIM-bearing Ig-like transcript 2 receptor suppresses B cell responses. J Immunol.

[45] Merlo A (2005). Inhibitory receptors CD85j, LAIR-1, and CD152 down-regulate immunoglobulin and cytokine production by human B lymphocytes. Clin Diagn Lab Immunol.

[46] Kim A (2019). LILRB1 blockade enhances bispecific T cell engager antibody–induced tumor cell killing by effector CD8+T cells. J Immunol.

[47] Lozano E (2018). Loss of the immune checkpoint CD85j/LILRB1 on malignant plasma cells contributes to immune escape in multiple myeloma. J Immunol.

[48] Dhodapkar MV (2016). MGUS to myeloma: a mysterious gammopathy of underexplored significance. Blood.

[49] Das R (2016). Microenvironment-dependent growth of preneoplastic and malignant plasma cells in humanized mice. Nat Med.

[50] Bray F (2018). Global cancer statistics 2018: GLOBOCAN estimates of incidence and mortality worldwide for 36 cancers in 185 countries. CA Cancer J Clin.

[51] Li Q (2020). Overexpressed immunoglobulin-like transcript (ILT) 4 in lung adenocarcinoma is correlated with immunosuppressive T cell subset infiltration and poor patient outcomes. Biomark Res.

[52] Deng M (2014). A motif in LILRB2 critical for Angptl2 binding and activation. Blood.

[53] Zheng J (2012). Inhibitory receptors bind ANGPTLs and support blood stem cells and leukaemia development. Nature.

[54] Liu X (2015). ANGPTL2/LILRB2 signaling promotes the propagation of lung cancer cells. Oncotarget.

[55] Wang L (2015). Co-expression of immunoglobulin-like transcript 4 and angiopoietin-like proteins in human non-small cell lung cancer. Mol Med Rep.

[56] Zhang P (2015). ILT4 drives B7-H3 expression via PI3K/AKT/mTOR signalling and ILT4/B7-H3 co-expression correlates with poor prognosis in non-small cell lung cancer. FEBS Lett.

[57] Chen H-M (2018). Blocking immunoinhibitory receptor LILRB2 reprograms tumor-associated myeloid cells and promotes antitumor immunity. J Clin Invest.

[58] Cai Z (2019). Immunoglobulin‑like transcript 4 and human leukocyte antigen‑G interaction promotes the progression of human colorectal cancer. Int J Oncol.

[59] Rouas-Freiss N (2017). Intratumor heterogeneity of immune checkpoints in primary renal cell cancer: focus on HLA-G/ILT2/ILT4. Oncoimmunology.

[60] Ristich V (2007). Mechanisms of prolongation of allograft survival by HLA-G/ILT4-modified dendritic cells. Hum Immunol.

[61] Apps R (2008). A critical look at HLA-G. Trends Immunol.

[62] Gao A (2021). Tumor-derived ILT4 induces T cell senescence and suppresses tumor immunity. J Immunother Cancer.

[63] Siu LL (2020). 5240 initial results of a phase I study of MK-4830, a first-in-class anti-immunoglobulin-like transcript 4 (ILT4) myeloid-specific antibody in patients with advanced solid tumours. Ann Oncol.

[64] https://clinicaltrials.gov/ct2/show/study/NCT03564691.

[65] https://ClinicalTrials.gov/show/NCT04669899.

[66] Jones DC (2016). Allele-specific recognition by LILRB3 and LILRA6 of a cytokeratin 8-associated ligand on necrotic glandular epithelial cells. Oncotarget.

[67] Gamrekelashvili J (2007). Necrotic tumor cell death in vivo impairs tumor-specific immune responses. J Immunol.

[68] Sporn MB (1996). The war on cancer. Lancet.

[69] Li J (2021). ILT3 promotes tumor cell motility and angiogenesis in non-small cell lung cancer. Cancer Lett.

[70] Singh L (2020). ILT3 (LILRB4) promotes the immunosuppressive function of tumor-educated human monocytic myeloid-derived suppressor cells. Mol Cancer Res.

[71] Cortesini R (2007). Pancreas cancer and the role of soluble immunoglobulin-like transcript 3 (ILT3). JOP.

[72] Suciu-Foca N (2007). Soluble Ig-like transcript 3 inhibits tumor allograft rejection in humanized SCID mice and T cell responses in cancer patients. J Immunol.

[73] Xu Z (2018). ILT3.Fc-CD166 interaction induces inactivation of p70 S6 kinase and inhibits tumor cell growth. J Immunol.

[74] Munitz A (2010). Inhibitory receptors on myeloid cells: new targets for therapy?. Pharmacol Ther.

[75] Döhner H (2015). Acute myeloid leukemia. N Engl J Med.

[76] Churchill HRO (2021). Leukocyte immunoglobulin-like receptor B1 and B4 (LILRB1 and LILRB4): highly sensitive and specific markers of acute myeloid leukemia with monocytic differentiation. Cytometry B Clin Cytom.

[77] Dobrowolska H (2013). Expression of immune inhibitory receptor ILT3 in acute myeloid leukemia with monocytic differentiation. Cytometry B Clin Cytom.

[78] Gui X (2019). Disrupting LILRB4/APOE interaction by an efficacious humanized antibody reverses T-cell suppression and blocks AML development. Cancer Immunol Res.

[79] John S (2018). A novel anti-LILRB4 CAR-T cell for the treatment of monocytic AML. Mol Ther.

[80] Anami Y (2020). LILRB4-targeting antibody-drug conjugates for the treatment of acute myeloid leukemia. Mol Cancer Ther.

[81] Deng M (2018). LILRB4 signalling in leukaemia cells mediates T cell suppression and tumour infiltration. Nature.

[82] Li Z (2020). LILRB4 ITIMs mediate the T cell suppression and infiltration of acute myeloid leukemia cells. Cell Mol Immunol.

[83] Wu G (2021). LILRB3 supports acute myeloid leukemia development and regulates T-cell antitumor immune responses through the TRAF2–cFLIP–NF-κB signaling axis. Nat Cancer.

[84] https://ClinicalTrials.gov/show/NCT04372433.

[85] Cai H (2020). Immune checkpoints in viral infections. Viruses.

[86] Meylan E (2006). Intracellular pattern recognition receptors in the host response. Nature.

[87] Akira S (2006). Pathogen recognition and innate immunity. Cell.

[88] Kawai T, Akira S (2010). The role of pattern-recognition receptors in innate immunity: update on Toll-like receptors. Nat Immunol.

[89] Brown DP (2009). The inhibitory receptor LILRB4 (ILT3) modulates antigen presenting cell phenotype and, along with LILRB2 (ILT4), is upregulated in response to Salmonella infection. BMC Immunol.

[90] Abdallah F (2021). Leukocyte immunoglobulin-like receptors in regulating the immune response in infectious diseases: a window of opportunity to pathogen persistence and a sound target in therapeutics. Front Immunol.

[91] Goel S (2015). RIFINs are adhesins implicated in severe Plasmodium falciparum malaria. Nat Med.

[92] Harrison TE (2020). Structural basis for RIFIN-mediated activation of LILRB1 in malaria. Nature.

[93] Sakoguchi A (2021). Plasmodium falciparum RIFIN is a novel ligand for inhibitory immune receptor LILRB2. Biochem Biophys Res Commun.

[94] Saito F (2017). Immune evasion of Plasmodium falciparum by RIFIN via inhibitory receptors. Nature.

[95] Nakayama M (2007). Paired Ig-like receptors bind to bacteria and shape TLR-mediated cytokine production. J Immunol.

[96] Hogan LE (2016). Expression of the innate immune receptor LILRB5 on monocytes is associated with mycobacteria exposure. Sci Rep.

[97] Ming S (2019). Immunoglobulin-like transcript 5 inhibits macrophage-mediated bacterial killing and antigen presentation during sepsis. J Infect Dis.

[98] Van Der Poll T, Opal SM (2008). Host-pathogen interactions in sepsis. Lancet Infect Dis.

[99] Zhao Y (2020). The orphan immune receptor LILRB3 modulates Fc receptor-mediated functions of neutrophils. J Immunol.

[100] Park M (2016). Leukocyte immunoglobulin-like receptor B4 regulates key signalling molecules involved in FcγRI-mediated clathrin-dependent endocytosis and phagocytosis. Sci Rep.

[101] Chan KR (2014). Leukocyte immunoglobulin-like receptor B1 is critical for antibody-dependent dengue. Proc Natl Acad Sci U S A.

[102] Ong EZ (2017). Dengue virus compartmentalization during antibody-enhanced infection. Sci Rep.

[103] Huang J (2010). Leukocyte immunoglobulin-like receptors maintain unique antigen-presenting properties of circulating myeloid dendritic cells in HIV-1-infected elite controllers. J Virol.

[104] Arnold V (2013). S100A9 protein is a novel ligand for the CD85j receptor and its interaction is implicated in the control of HIV-1 replication by NK cells. Retrovirology.

[105] Cosman D (1997). A novel immunoglobulin superfamily receptor for cellular and viral MHC class I molecules. Immunity.

[106] Prod’homme V (2007). The human cytomegalovirus MHC class I homolog UL18 inhibits LIR-1+ but activates LIR-1- NK cells. J Immunol.

[107] Saverino D (2004). Specific recognition of the viral protein UL18 by CD85j/LIR-1/ILT2 on CD8+ T cells mediates the non-MHC-restricted lysis of human cytomegalovirus-infected cells. J Immunol.

[108] Berg L (2003). LIR-1 expression on lymphocytes, and cytomegalovirus disease in lung-transplant recipients. Lancet.

[109] Marrack P (2001). Autoimmune disease: why and where it occurs. Nat Med.

[110] Rosenblum MD (2015). Mechanisms of human autoimmunity. J Clin Invest.

[111] Kim SY, Solomon DH (2010). Tumor necrosis factor blockade and the risk of viral infection. Nat Rev Rheumatol.

[112] Targownik LE, Bernstein CN (2013). Infectious and malignant complications of TNF inhibitor therapy in IBD. Am J Gastroenterol.

[113] Thomas R (2010). Leukocyte immunoglobulin-like receptors as new players in autoimmunity. Clin Rev Allergy Immunol.

[114] Xu Z (2020). Suppression of experimental autoimmune encephalomyelitis by ILT3.Fc. J Immunol.

[115] Van Der Touw W (2018). Glatiramer acetate enhances myeloid-derived suppressor cell function via recognition of paired Ig-like receptor B. J Immunol.

[116] Degani Veit T (2015). The paradox of high availability and low recognition of soluble HLA-G by LILRB1 receptor in rheumatoid arthritis patients. PLoS One.

[117] Shiroishi M (2006). Efficient leukocyte Ig-like receptor signaling and crystal structure of disulfide-linked HLA-G dimer. J Biol Chem.

[118] Gonzalez A (2011). Identification of circulating nonclassic human leukocyte antigen G (HLA-G)-like molecules in exudates. Clin Chem.

[119] Monsiváis-Urenda A (2007). Analysis of expression and function of the inhibitory receptor ILT2 (CD85j/LILRB1/LIR-1) in peripheral blood mononuclear cells from patients with systemic lupus erythematosus (SLE). J Autoimmun.

[120] Cervera R (1993). Systemic lupus erythematosus: clinical and immunologic patterns of disease expression in a cohort of 1,000 patients. The European Working Party on systemic lupus erythematosus. Medicine (Baltimore).

[121] Wandstrat AE (2006). Autoantibody profiling to identify individuals at risk for systemic lupus erythematosus. J Autoimmun.

[122] Wu J-F (2006). Antinucleosome antibodies correlate with the disease severity in children with systemic lupus erythematosus. J Autoimmun.

[123] Monsiváis-Urenda A (2013). Defective expression and function of the ILT2/CD85j regulatory receptor in dendritic cells from patients with systemic lupus erythematosus. Hum Immunol.

[124] Delgado De La Poza JF (2011). Contribution of LILRB1 polymorphism and HLA-DRB1-shared epitope to rheumatoid arthritis. Inmunología.

[125] Bergamini A (2014). Downregulation of immunoglobulin-like transcript-4 (ILT4) in patients with psoriatic arthritis. PLoS One.

[126] Hirayasu K (2021). Characterization of LILRB3 and LILRA6 allelic variants in the Japanese population. J Hum Genet.

[127] Renauer PA (2015). Identification of susceptibility loci in IL6, RPS9/LILRB3, and an intergenic locus on chromosome 21q22 in Takayasu arteritis in a genome-wide association study. Arthritis Rheumatol.

[128] Yoshifuji H, Terao C (2020). Roles of cytotoxic lymphocytes and MIC/LILR families in pathophysiology of Takayasu arteritis. Inflamm Regen.

[129] Farrar CA (2013). The innate immune system and transplantation. Cold Spring Harb Perspect Med.

[130] Shrestha BM (2017). Immune system and kidney transplantation. JNMA J Nepal Med Assoc.

[131] Zhang W (2008). Human inhibitory receptor immunoglobulin-like transcript 2 amplifies CD11b+Gr1+ myeloid-derived suppressor cells that promote long-term survival of allografts. Transplantation.

[132] Le Bouteiller P (2015). HLA-G in human early pregnancy: control of uterine immune cell activation and likely vascular remodeling. Biomed J.

[133] Roghanian A (2019). Cyclophosphamide enhances cancer antibody immunotherapy in the resistant bone marrow niche by modulating macrophage FcγR expression. Cancer Immunol Res.

[134] Ge G (2012). Induction of CD4+ CD25+ Foxp3+ T regulatory cells by dendritic cells derived from ILT3 lentivirus-transduced human CD34+ cells. Transpl Immunol.

[135] Vlad G (2008). Immunoglobulin-like transcript 3-Fc suppresses T-cell responses to allogeneic human islet transplants in hu-NOD/SCID mice. Diabetes.

[136] Vlad G (2009). Suppression of xenogeneic graft-versus-host disease by treatment with immunoglobulin-like transcript 3-Fc. Hum Immunol.

[137] Vlad G (2010). Membrane and soluble ILT3 are critical to the generation of T suppressor cells and induction of immunological tolerance. Int Rev Immunol.

[138] Vlad G (2011). Gene profile analysis of CD8(+) ILT3-Fc induced T suppressor cells. Hum Immunol.

[139] Naji A (2007). Soluble HLA-G and HLA-G1 expressing antigen-presenting cells inhibit T-cell alloproliferation through ILT-2/ILT-4/FasL-mediated pathways. Hum Immunol.

[140] Pfistershammer K (2009). Allogeneic disparities in immunoglobulin-like transcript 5 induce potent antibody responses in hematopoietic stem cell transplant recipients. Blood.

[141] Fan X (2014). Paired immunoglobulin-like receptor B regulates platelet activation. Blood.

[142] Takeda K, Nakamura A (2017). Regulation of immune and neural function via leukocyte Ig-like receptors. J Biochem.

